# Clinical characteristics and outcomes associated with preserved ratio impaired spirometry (PRISm) in Saudi Arabia

**DOI:** 10.3389/fmed.2026.1730537

**Published:** 2026-02-19

**Authors:** Abdullah A. Alqarni, Abdulelah M. Aldhahir, Hassan Alwafi, Rayan A. Siraj, Jaber S. Alqahtani, Hanan F. Nassier, Maria M. Kutbi, Joud S. Sager, Hanadi A. Balfas, Ahmed H. Alasimi, Yousef S. Aldabayan, John R. Hurst

**Affiliations:** 1 Department of Respiratory Therapy, Faculty of Medical Rehabilitation Sciences, King Abdulaziz University, Jeddah, Saudi Arabia; 2 Respiratory Therapy Unit, King Abdulaziz University Hospital, Jeddah, Saudi Arabia; 3 Respiratory Therapy Program, Department of Nursing, College of Nursing and Health Sciences, Jazan University, Jazan, Saudi Arabia; 4 Department of Clinical Pharmacology and Toxicology, Faculty of Medicine, Umm Al-Qura University, Makkah, Saudi Arabia; 5 Department of Respiratory Care, College of Applied Medical Sciences, King Faisal University, Al-Ahsa, Saudi Arabia; 6 Department of Respiratory Care, Prince Sultan Military College of Health Sciences, Dammam, Saudi Arabia; 7 National Heart and Lung Institute, Imperial College London, London, United Kingdom; 8 UCL Respiratory, Royal Free Campus, University College London, London, United Kingdom

**Keywords:** dyspnea, lung function, preserved ratio impaired spirometry, PRISm, quality of life, respiratory symptoms, spirometry

## Abstract

**Background:**

Preserved ratio impaired spirometry (PRISm) is an abnormal spirometric pattern associated with increased morbidity and mortality. However, its psychological and symptomatic burden remains poorly characterized. This study aimed to: (1) assess the prevalence of anxiety, depression, breathlessness, impaired health status, and reduced quality of life; (2) evaluate the impact of psychological and respiratory symptoms on clinical outcomes; and (3) explore the associations of psychological and respiratory symptoms with clinical outcomes among patients with PRISm in Saudi Arabia.

**Methods:**

Breathlessness was assessed using the modified Medical Research Council (mMRC) Dyspnea Scale. Symptoms of anxiety and depression were evaluated using the Hospital Anxiety and Depression Scale (HADS). Quality of life was measured using the St. George’s Respiratory Questionnaire (SGRQ). Overall health status and the impact of respiratory symptoms on daily activities were assessed using the Chronic Airways Assessment Test (CAAT).

**Results:**

A total of 101 patients with PRISm met our inclusion criteria and were included in the analysis. Of these patients, 38 (37.6%) exhibited symptoms of anxiety, and 27 (26.7%) exhibited symptoms of depression. Furthermore, 45 (44.5%) patients exhibited impacts on their health status in association with PRISm, 37 (36.6%) had increased levels of breathlessness, and 67 (66.3%) had impaired quality of life. PRISm patients with uncontrolled respiratory symptoms have reduced health status and increased levels of psychological symptoms compared with those with controlled symptoms. In addition, quality of life, health status, and respiratory symptoms were significantly impaired in patients with depressive or anxious symptoms compared with those without depression or anxiety. Although no associations were observed with hospital-based outcomes, depression was associated with a higher number of comorbidities.

**Conclusion:**

Our study has shown that individuals with PRISm face substantial respiratory and psychological difficulties, including elevated anxiety and depression levels, as well as frequent hospitalizations. Given that PRISm is underdiagnosed and underappreciated with no clear guidelines on treatment plans, these findings underscore the critical need for routine assessments and comprehensive management strategies to enhance the quality of life for PRISm patients.

## Introduction

Preserved ratio impaired spirometry (PRISm) has emerged as a significant clinical entity within the realm of pulmonary medicine, characterized by a distinct spirometric profile that diverges from traditional obstructive and restrictive lung diseases (1). Specifically, PRISm is defined by a reduced forced expiratory volume in 1 s (FEV1) of less than 80% of the predicted value while maintaining a normal FEV1/forced vital capacity (FVC) ratio of ≥ 0.7 (2). This unique phenotype is often overlooked in clinical practice, as it does not meet the diagnostic criteria for chronic obstructive pulmonary disease (COPD); however, it is associated with notable morbidity and mortality outcomes (3, 4). The prevalence of PRISm varies widely across populations, with estimates ranging from 3 to 22% in adults (5). Notably, PRISm has been observed more frequently in individuals with a history of smoking, in those with higher body mass indices (BMIs), and in certain demographic groups, such as women and older adults (6–8).

PRISm has garnered increasing attention in recent years due to its association with various adverse health outcomes, including respiratory symptoms, comorbidities, and mortality (4, 9). Epidemiological studies indicate that individuals with PRISm often report elevated levels of psychological disorders, such as depression and anxiety, which can be exacerbated by the stress of managing a chronic illness (9, 10). Previous evidence has revealed that individuals with PRISm experience a higher burden of respiratory symptoms and a decline in health-related quality of life than those with normal spirometry (11, 12). This deterioration in quality of life is particularly concerning given the potential for PRISm to progress to more severe respiratory diseases, including COPD, which further complicates patients’ daily functioning and overall health status (13, 14).

Importantly, the presence of PRISm has been associated with an increased risk of developing COPD over time, highlighting the need for vigilant monitoring and management of affected individuals (5). Furthermore, the heterogeneity of PRISm necessitates a nuanced approach to its assessment, as recent evidence has identified subtypes that may exhibit distinct clinical trajectories and outcomes (15). As such, the lack of standardized diagnostic criteria and the tendency for PRISm to be misclassified as normal or obstructive lung function can lead to underdiagnosis and inadequate treatment (16). Consequently, patients with PRISm may experience increased respiratory symptoms, reduced quality of life, and increased healthcare utilization as their condition progresses (11, 17). The need for a comprehensive understanding of PRISm is further emphasized by its association with adverse health outcomes, including increased hospitalization and mortality rates (7).

Despite its clinical significance, PRISm has historically been underrecognized and under-researched compared to other pulmonary conditions. This oversight may be attributed to the lack of standardized diagnostic criteria and the tendency to focus on obstructive lung diseases in clinical practice (6). Given the absence of current management guidelines and the limited available data on the clinical characteristics of individuals with PRISm, this study aimed to: (1) describe the prevalence of anxiety, depression, breathlessness, overall health status, and quality of life; (2) examine differences in clinical outcomes according to psychological and respiratory symptom burden; and (3) explore the associations of psychological and respiratory symptoms with clinical outcomes among patients with PRISm in Saudi Arabia.

## Methods

### Study design and setting

This was a hospital-based, cross-sectional study carried out between November 2023 and October 2024 at King Abdulaziz University Hospital, Jeddah, Saudi Arabia.

### Study population

The study population consisted of patients attending the pulmonary clinic with respiratory symptoms. The inclusion criteria included patients who were able to perform spirometry and who provided their informed consent to participate. The exclusion criteria included patients who were under 18 years old, could not meet acceptable and reproducible spirometry criteria, and refused to participate in the study. In addition, patients with a prior diagnosis of other respiratory diseases, including asthma and alpha-1 antitrypsin deficiency, were excluded to minimize diagnostic overlap and potential confounding.

### Spirometry parameters

We defined individuals with PRISm only if they had a post-bronchodilator FEV1 of less than 80% predicted and a post-bronchodilator FEV1/FVC of 0.70 or more, using the Global Lung Initiative Mixed Ethnic reference values (18). Spirometry tests were performed using a SensorMedics Vmax 22 machine (SensorMedics Inc., Anaheim, CA, USA) by a certified pulmonary function technician. To further validate the tests, a pulmonologist (M. S. D.) and two consultant respiratory therapists (A. A. A. and A. M. A.) further reviewed the tests and confirmed that they were performed in accordance with the current American Thoracic Society/European Respiratory Society guidelines (19). Those who were unable to meet the acceptable and reproducible criteria for spirometry were scheduled to perform the test on a different date and were subsequently excluded if the test remained unacceptable.

### Data collection procedures

Data from individuals with acceptable and reproducible spirometry test results were collected by four respiratory therapy interns (H. F. N., M. M. K., J. S. S., and H. A. B.) under the supervision of two respiratory therapy consultants (A. A. A. and A. M. A.), one senior respiratory therapy specialist (R. A. S.), and a pulmonologist. Prior to this, the study objectives were clearly explained to individuals, and only those who provided informed consent were included in the study.

### Patient-reported outcome measures

Participants who agreed to take part underwent interviews and were asked to complete four assessment tools: the Hospital Anxiety and Depression Scale (HADS), the St. George’s Respiratory Questionnaire (SGRQ), the Chronic Airways Assessment Test (CAAT), and the modified Medical Research Council (mMRC) dyspnea scale, as described below. The validity of these tools has been previously established within the Arab population (20–23). All other demographic and clinical characteristics of all patients were then collected from their medical records.

#### Anxiety and depression

The HADS is a 14-item tool used to identify and quantify the levels of anxiety and depression among hospital patients who may require further psychiatric intervention and assistance. It is used to establish an initial diagnosis and monitor symptoms during therapy. The HADS consists of 14 questions, divided into 7 for depression and 7 for anxiety. Each response is scored from 0 to 3, with 0 indicating no impairment and 3 severe impairments. Therefore, the total score ranges from 0 to 21. A score of 11–21 suggests abnormalities, while a score of 7 or below indicates a normal degree of psychological symptoms. Scores between 0 and 1 are considered borderline (24).

#### Quality of life

The SGRQ was used in the current study to assess the quality of life among individuals with PRISm. The SGRQ consists of 50 items that address three distinct domains: symptoms, activity, and impact. The symptoms domain assesses the frequency and severity of respiratory symptoms, while the activity domain evaluates activities that are either caused or limited by dyspnea. The impact domain reflects the effects of respiratory diseases on psychosocial functioning. Scores range from 0 to 100, with lower scores indicating better health-related quality of life and higher scores indicating greater impairment (25). In this study, we used an SGRQ total score ≥ 25 to define impairment in quality of life.

#### Respiratory symptoms

The mMRC dyspnea scale is an assessment tool used to measure breathlessness in patients with pulmonary disease. The mMRC scale is simple, rapid, and can be self-administered by asking the patient to select only one statement or phrase that best describes their condition. It is rated on a scale from Grade 0 to Grade 4. Grade 0 indicates breathlessness only with strenuous exercise, while Grade 1 indicates shortness of breath when hurrying on level ground or walking up a slight hill. Grade 2 describes individuals who walk more slowly than people of the same age due to breathlessness or who must stop to catch their breath when walking at their own pace. Grade 3 refers to individuals who stop for breath after walking approximately 100 meters or after a few minutes on level ground. Grade 4 indicates severe breathlessness that prevents leaving the house or causes breathlessness during dressing or undressing. In the current study, an mMRC score of 2–4 was used to define increased breathlessness (26). Participants were categorized into two groups based on the severity of respiratory symptoms: controlled symptoms (mMRC dyspnea scale score 0–1) and uncontrolled symptoms (mMRC dyspnea scale score 2–4). This classification was used for subsequent comparisons of psychological symptoms, health status, and quality of life.

#### Overall health status

The CAAT, also known as the COPD Assessment Test (CAT), was used to assess the overall health status and the impact of respiratory symptoms, including cough, sputum production, dyspnea, shortness of breath, and limitations in daily activities. The CAAT consists of eight items, with responses scored on a scale from 0 to 5. Higher scores reflect a greater impact of respiratory symptoms on health status and quality of life and assist healthcare professionals in developing appropriate care plans (27). In the current study, the medium-to-high impact of respiratory symptoms on health status was defined as a CAAT score of 20–40.

### Ethical considerations

Before the start of this study, we obtained ethical approval from the Research Ethics Committee at King Abdulaziz University Hospital, Jeddah, Saudi Arabia (No. 457-23).

### Statistical analysis

GraphPad Prism (Version 9) was used to analyze the data. Data normality was assessed visually through histograms to inform the choice of statistical tests, as formal normality tests can be overly sensitive in moderate sample sizes and were not required for the analysis. Categorical variables were expressed as frequencies and percentages, while continuous variables with a normal distribution were reported as mean values ± standard deviations.

The patients were divided into two groups based on the severity of respiratory symptoms: controlled and uncontrolled. Then, the levels of depression, anxiety, quality of life, and health status were assessed. We also divided participants into two groups based on the severity of their psychological symptoms to assess whether quality of life, health status, and respiratory symptoms are impaired in PRISm patients with anxious and depressive symptoms. Group differences were assessed using unpaired t-tests with Welch’s correction for two-group comparisons, and effect sizes (Cohen’s d) were calculated to quantify the magnitude of differences. We then performed regression analyses to determine the factors associated with psychological and respiratory symptoms among those with PRISm. Multivariable regression models were adjusted *a priori* for age, sex, BMI, and smoking status based on their established clinical relevance and prior literature. A *p*-value *of <* 0.05 was considered statistically significant.

## Results

### Patient characteristics

**Figure 1:**
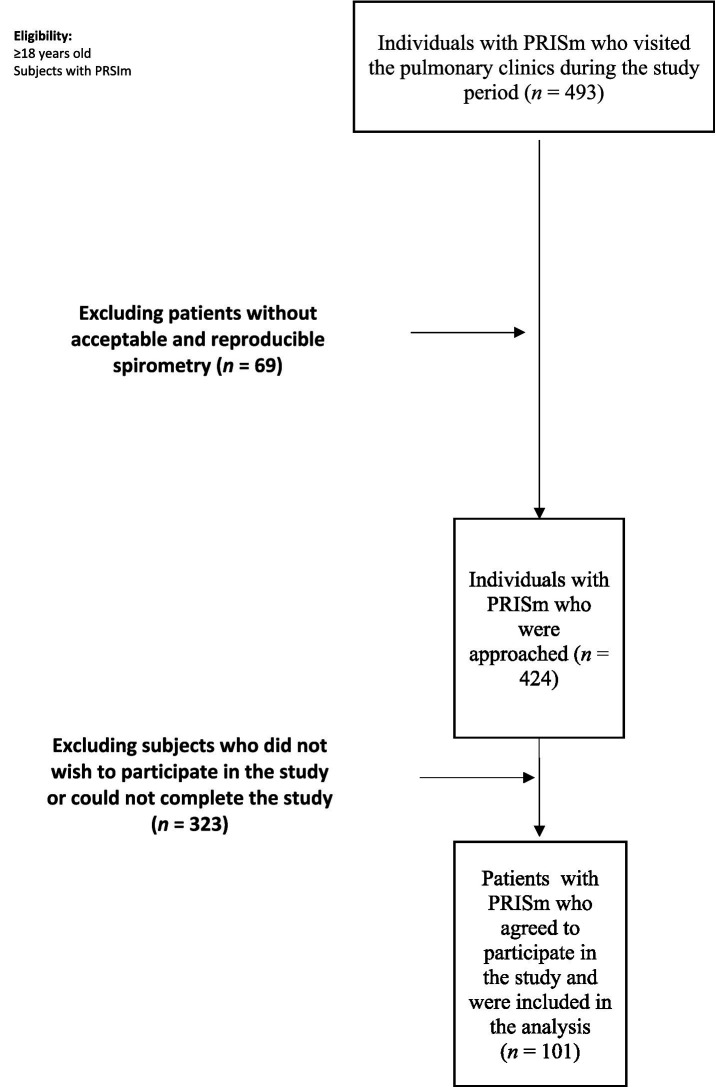
Flowchart of the study.

A total of 493 patients with PRISm visited the pulmonary clinics for routine follow-up during the data collection process. Of these, 101 patients met our inclusion criteria and were included in the final analysis (Figure 1).

The mean ± SD age and body mass index (BMI) of our study population were 52 ± 14 years and 30 ± 6.9 kg/m2, respectively. As shown in Table 1, 74% of our study population had one or more comorbidities, and almost half of our subjects had been infected with COVID-19, 45.5% had a hospital admission, and 14% were admitted to the ICU in the last year due to worsening respiratory symptoms. Other demographic and clinical characteristics of the study participants are described in Table 1.

**Table 1:** Patient characteristics.

Variable	PRISm (*n* = 101)
Age (years)	52 ± 14
Male, *n* (%)	40 (39.6%)
BMI (kg/m^2^)	30 ± 6.9
History of smoking	
No, *n* (%)	74 (73.2%)
Yes, *n* (%)	27 (26.7%)
Exposure to biomass for more than 1 year	
No, *n* (%)	86 (85.1%)
Yes, *n* (%)	15 (14.8%)
Occupational history with exposure to dusts/gases/fumes for more than 1 year	
No, *n* (%)	84 (83.1%)
Yes, *n* (%)	17 (16.8%)
Previously infected with COVID-19	
No, *n* (%)	52 (51.5%)
Yes, *n* (%)	49 (48.5%)
Number of comorbidities	
None, *n* (%)	25 (24.7%)
One, *n* (%)	35 (34.6%)
Two or more, *n* (%)	41 (40.0%)
Number of inhaled medications	
None, *n* (%)	30 (29.7%)
One, *n* (%)	31 (30.6%)
Two or more, *n* (%)	40 (39.6%)
Hospital admission due to worsening respiratory symptoms in the last year	
No, *n* (%)	55 (54.4%)
Yes, *n* (%)	46 (45.5%)
ICU admission due to worsening respiratory symptoms in the last year	
No, *n* (%)	87 (86.1%)
Yes, *n* (%)	14 (13.8%)
Spirometry parameters (post)	
FEV_1_ (% predicted)	64 ± 13
FVC (% predicted)	60 ± 14
FEV_1_/FVC ratio	0.82 ± 0.07

Data are represented as the mean ± SD unless otherwise stated. BMI, body mass index; ICU, intensive care unit; FVC, forced vital capacity; FEV_1_, forced expiratory volume in 1 s; COVID-19, coronavirus disease 2019.

### The prevalence of anxiety, depression, breathlessness, overall health status, and quality of life among patients with PRISm

Among the 101 patients included in the study, 38 (37.6, 95% CI: 28.6 to 47.5) and 27 (26.7, 95% CI: 18.9 to 36.3) patients had symptoms of anxiety and depression, respectively. The levels of health status impairment and breathlessness among our study population were 45 (44.5, 95% CI 34.5 to 53.9) and 37 (36.6, 95% CI 27.1 to 45.9), respectively, and 67 (66.3, 95% CI 56.4 to 74.9) patients had impaired quality of life.

### Anxiety, depression, quality of life, and health status in PRISm patients with uncontrolled respiratory symptoms

**Figure 2:**
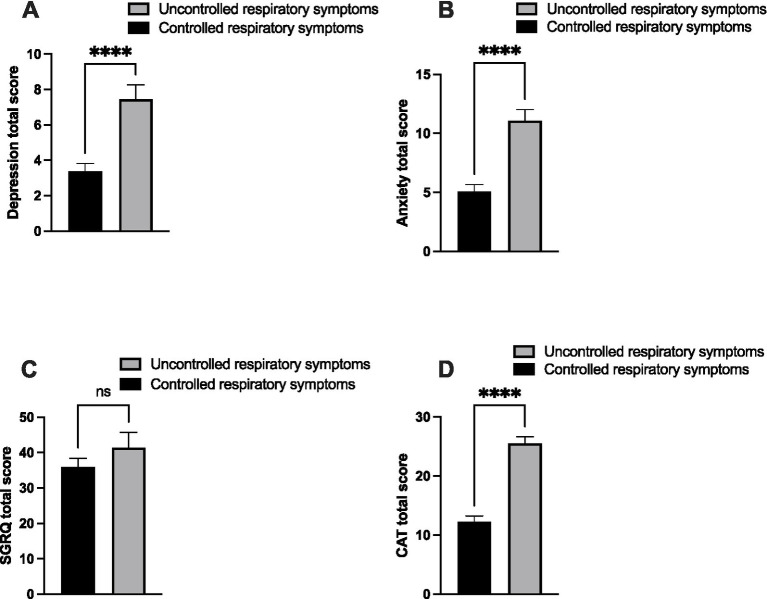
Assessment of depression, anxiety, health status, and quality of life among PRISm patients with uncontrolled respiratory symptoms. Patients were divided into two groups based on the severity of respiratory symptoms: controlled (Modified Medical Research Council (mMRC) score of 0–1, *n* = 64) and uncontrolled (mMRC score of 2–4, *n* = 37). Then, the levels of depression and anxiety were assessed using the Hospital Anxiety and Depression Scale (HADS) **(A,B)**. The levels of quality of life and health status were assessed using the St. George’s Respiratory Questionnaire (SGRQ) and the Chronic Airways Assessment Test (CAAT), respectively **(C,D)**. The bars represent the standard errors of the mean value *p* < 0.0001 compared with the controlled respiratory symptoms.

We next compared psychological symptoms, quality of life, and health status between PRISm patients with controlled and uncontrolled respiratory symptoms. Depression scores were significantly lower in patients with controlled respiratory symptoms than those with uncontrolled symptoms (3.16 ± 0.45 vs. 7.19 ± 0.74; *p* < 0.001), with a large effect size (Cohen’s d = 1.02; 95% CI: 0.59–1.45) (Figure 2A). Similarly, anxiety scores were significantly lower among patients with controlled symptoms than those with uncontrolled symptoms (4.94 ± 0.61 vs. 10.38 ± 0.84; *p* < 0.001), also demonstrating a large effect size (Cohen’s d = 1.10; 95% CI: 0.66–1.53) (Figure 2B).

We then examined the impact of poor respiratory symptom control on quality of life. Although the mean SGRQ was higher in patients with poorly controlled symptoms (38.8 ± 3.8) than those with controlled symptoms (36.95 ± 2.6), this difference was not statistically significant (*p* = 0.672) (Figure 2C). In contrast, CAT scores were significantly elevated in patients with poorly controlled symptoms (24.14 ± 1.10) than those with controlled symptoms (11.84 ± 1.04; *p* < 0.001), with a very large effect size (Cohen’s d = 1.59; 95% CI: 1.12–2.05) (Figure 2D). These results indicate that PRISm subjects with uncontrolled respiratory symptoms exhibit greater psychological symptom burden and reduced overall health status than those with controlled symptoms.

### Quality of life, health status, and breathlessness in PRISm patients with anxious and depressive symptoms

**Figure 3:**
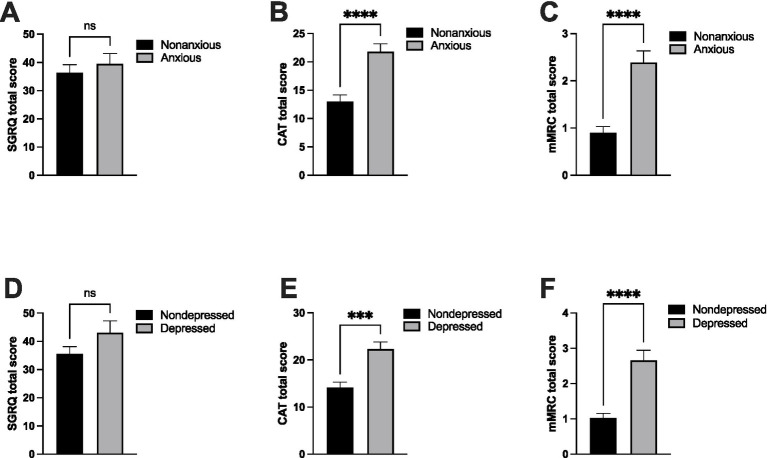
Assessment of quality of life, health status, and breathlessness in PRISm patients with anxious and depressive symptoms. The patients were divided into two groups based on the severity of psychological symptoms. Patients with Hospital Anxiety and Depression Scale (HADS) scores of 8 to 21 were classified as anxious (*n* = 38) and depressed (*n* = 27). HADS scores of 0 to 7 were considered non-anxious (*n* = 63) or non-depressed (*n* = 74). The levels of quality of life, health status, and breathlessness in PRISm patients were then assessed. The levels of quality of life, health status, and respiratory symptoms were assessed using the St. George’s Respiratory Questionnaire (SGRQ) **(A,D)**, the Chronic Airways Assessment Tool (CAAT) **(B,E)**, and the modified Medical Research Council (mMRC) **(C,F)**, respectively. The bars represent the standard errors of the mean value s***p < 0.001 and ****p < 0.0001 compared with non-anxious or non-depressed.

We next divided the patients into two groups based on the severity of their psychological symptoms to assess whether quality of life, health status, and respiratory symptoms are associated with anxiety and depression in PRISm. The mean SGRQ was slightly higher among anxious patients than among non-anxious patients (39.6 ± 3.6 vs. 36.5 ± 2.7); however, this difference was not statistically significant (*p* = 0.493) (Figure 3A). In contrast, CAT scores were significantly elevated in anxious patients (21.8 ± 1.4) than in non-anxious patients (13.0 ± 1.1; *p* < 0.001), with a large effect size (Cohen’s d = 1.00; 95% CI 0.58–1.43) (Figure 3B). Similarly, mMRC scores were higher in anxious patients (2.4 ± 0.2) than in non-anxious patients (0.9 ± 0.1; *p* < 0.001), also demonstrating a large effect size (Cohen’s d = 1.21; 95% CI 0.79–1.67) (Figure 3C). These findings indicate that PRISm patients with anxiety are more likely to experience impaired health status and increased respiratory symptoms.

We then evaluated the impact of depressive symptoms. There were no statistically significant differences in SGRQ scores between depressed and non-depressed patients (43.1 ± 4.1 vs. 35.6 ± 2.5; *p* = 0.121) (Figure 3D). However, CAT scores were significantly higher in depressed patients (22.3 ± 1.5) than in non-depressed patients (14.2 ± 1.1; *p* < 0.001), with a large effect size (Cohen’s d = 0.90; 95% CI 0.44–1.36) (Figure 3E), indicating reduced overall health status. In addition, mMRC scores were elevated in depressed patients (2.7 ± 0.3) than in non-depressed patients (1.0 ± 0.1; *p* < 0.001), with a large effect size (Cohen’s d = 1.36; 95% CI 0.88–1.84) (Figure 3F), reflecting greater breathlessness in patients with depressive symptoms.

### Associations of psychological and respiratory symptoms with clinical outcomes among patients with PRISm

We next assessed the associations of psychological and respiratory symptoms with clinical outcomes among patients with PRISm. We found no associations between respiratory symptoms, anxiety, and quality of life with ICU/hospital admission and number of comorbidities among patients with PRISm (Table 2). Although we found no associations between depression and quality of life and ICU/hospital admission, depression was associated with the number of comorbidities among patients with PRISm.

**Table 2:** Analysis of the associations of psychological and respiratory symptoms with clinical outcomes among patients with PRISm (*n* = 101).

Clinical outcomes	Anxiety score	Depression score	mMRC score
	^†^Adjusted β (95% CI)	^†^Adjusted β (95% CI)	^†^Adjusted β (95% CI)
SGRQ total score	0.02 (−0.03 to 0.07)	0.01 (−0.02 to 0.06)	0.00 (−0.00 to 0.02)
ICU admission	−1.0 (−4.23 to 2.20)	−1.86 (−4.39 to 0.67)	−0.36 (−1.17 to 0.44)
Hospital admission	0.62 (−1.58 to 2.83)	−0.31 (−2.07 to 1.44)	−0.22 (−0.78 to 0.33)
Number of comorbidities	0.37 (−0.15 to 1.90)	1.54 (0.36 to 2.72) *	0.23 (−0.15 to 0.61)

† Adjusted for age, sex, smoking status, and body mass index, *p < 0.05.

SGRQ, St. George’s Respiratory Questionnaire; mMRC, modified Medical Research Council’s dyspnea scale; ICU, intensive care unit.

## Discussion

This study explored the prevalence of anxiety, depression, breathlessness, overall health status, and quality of life among individuals with PRISm in Saudi Arabia and evaluated the impact of psychological and respiratory symptoms on clinical outcomes. Our findings revealed that individuals with PRISm face substantial respiratory and psychological difficulties, with a considerable proportion of patients experiencing anxiety and depressive symptoms, impaired quality of life, reduced health status, and breathlessness. Importantly, individuals with uncontrolled respiratory symptoms had poorer health status and higher levels of anxiety and depression compared with those with controlled symptoms. Although no associations were observed with hospital-based outcomes, depression was associated with a higher number of comorbidities.

Research consistently documents that anxiety and depression exacerbate chronic respiratory conditions, with an intricate link between psychological distress and respiratory symptoms (28–30). In this study, 37.6% of the participants experienced anxiety symptoms, while 26.7% reported depressive symptoms. Although psychological symptoms have not been assessed previously in individuals with PRISm, our findings are consistent with previous literature demonstrating a high prevalence of emotional disturbance among individuals with chronic respiratory diseases (31, 32). Psychological burden in respiratory disease populations has been attributed to functional limitations, symptom unpredictability, and activity restriction, which collectively contribute to reduced quality of life (10). Longitudinal studies have shown the significant effect of psychological distress on pulmonary health, indicating that patients experiencing anxiety and depression are at increased risk for acute exacerbations, declining lung function, and even higher mortality (10, 33). This underscores the necessity of incorporating psychological assessments as part of routine evaluations for patients exhibiting respiratory symptoms, including those diagnosed with PRISm (16).

Additionally, the health status impairment (44.5%) and breathlessness (36.6%) observed in this study have shown that PRISm is not merely an incidental spirometric pattern but is frequently accompanied by clinically meaningful symptoms. These results align with previous studies suggesting that traditional spirometric indices alone fail to capture the symptom burden experienced by individuals with PRISm (34, 35). Subsequent literature has shown that PRISm is associated with a broader spectrum of adverse health outcomes, including impaired health-related quality of life and increased respiratory symptoms (5, 36). Breathlessness, a dominant symptom in chronic respiratory disease, is known to limit physical activity and reduce quality of life (37). Although mechanistic pathways cannot be established from our data, prior studies have suggested that breathlessness in PRISm may reflect a combination of reduced ventilatory reserve, altered lung mechanics, obesity-related restriction, and coexisting psychological distress (11, 38). Notably, individuals with PRISm have been reported to experience worse respiratory health status than those with mild COPD (GOLD stage I) (34). Furthermore, a previous study has indicated that breathlessness may be a stronger determinant of health status than spirometric severity (39). These findings highlight the need to evaluate symptoms and functional status alongside lung function when assessing PRISm.

The pervasive impairment in quality of life observed in 66.3% of participants underscores the significant impact of PRISm on overall wellbeing. Comparable reductions in health-related quality of life have been reported in PRISm populations in prior studies, despite preserved FEV₁/FVC ratios (4, 6, 11). Notably, previous studies have suggested that this impairment is driven by persistent breathlessness, reduced physical capacity, and psychological comorbidities rather than airflow limitation alone (11, 40). Furthermore, systemic inflammation has been proposed as a contributing factor linking PRISm with both physical and psychological symptoms; however, this mechanism was not assessed in the current study and should be interpreted cautiously (8, 41). These observations emphasize the multifactorial nature of quality-of-life impairment in PRISm and suggest that management strategies should extend beyond spirometric classification.

A key finding of this study is that PRISm participants with uncontrolled respiratory symptoms exhibited poorer health status and greater psychological distress than those with controlled symptoms. Similar associations between worsening health status and increased vulnerability to anxiety and depression have been reported in other respiratory populations (42, 43). Prior studies have suggested that anxiety may amplify symptom perception and contribute to activity avoidance, thereby accelerating functional decline (15, 36). Likewise, depressive symptoms have been linked to reduced treatment adherence, higher symptom burden, and poorer overall health outcomes in chronic respiratory disease (44–46). While our study cannot establish causality, the observed coexistence of uncontrolled respiratory symptoms and psychological distress suggests a bidirectional relationship that warrants integrated clinical assessment (47). Recognizing PRISm as a distinct and heterogeneous clinical entity has prompted calls for its inclusion in clinical guidelines and future research frameworks, as individuals with this condition often remain underdiagnosed and undertreated (48). Part of the challenge here is the heterogeneity of people with PRISm, and a greater understanding is required of different phenotypes in PRISm.

### Strengths and limitations

This study is the first to assess the clinical outcomes associated with PRISm patients in Saudi Arabia. The use of appropriate and validated instruments to assess respiratory symptoms, psychological distress, and overall health status lends credibility to the findings. Additionally, including clinical outcomes, such as hospitalization and ICU admission, strengthens the study’s clinical relevance by highlighting the real-world burden of PRISm. However, several limitations should be acknowledged. The cross-sectional design precludes causal inference. Of the 493 initially identified patients, only 101 were included, limiting statistical power and subgroup analyses. The high rate of refusals and losses (>75% of potentially eligible participants) introduces a risk of selection bias, and the absence of comparative data on excluded patients may limit representativeness. This high non-participation rate may be partly due to the time required to complete multiple questionnaires and reluctance to disclose psychological symptoms. Psychological/respiratory symptoms and quality of life were assessed using self-reported questionnaires, which may be subject to reporting bias. Several relevant potential confounders—including socioeconomic status, physical activity, treatment adherence, and inflammatory biomarkers—were not assessed. In addition, PRISm was defined using fixed-ratio criteria, although lower limits of normal (LLN)-based definitions may classify some individuals differently. As this was an exploratory study, no *a priori* sample size calculation was performed. Finally, the relatively small sample size, single-center design, inclusion of younger adults, and lack of respiratory muscle strength assessment may limit the generalizability and mechanistic interpretation of the findings, particularly in older PRISm populations.

## Conclusion

This study elucidates the substantial burden of PRISm on patients’ respiratory health, psychological wellbeing, and overall clinical outcomes in Saudi Arabia. A considerable proportion of patients experienced anxiety, depression, breathlessness, and impaired quality of life, emphasizing the need for increased awareness and early identification of PRISm in clinical settings. Given the underdiagnosed and underappreciated nature of PRISm, the development of standardized treatment guidelines and routine screening for psychological symptoms should be prioritized to improve patient care and outcomes. Future longitudinal studies are warranted to further explore the progression of PRISm and its long-term impact on health and quality of life.

## Data Availability

The raw data supporting the conclusions of this article will be made available by the authors, without undue reservation.
