# Supplementary material for: Clinical characteristics and outcomes associated with preserved ratio impaired spirometry (PRISm) in Saudi Arabia
Source: Front Med (Lausanne). 2026 Feb 19;13:1730537. doi: 10.3389/fmed.2026.1730537 (PMC12961682; doi:10.3389/fmed.2026.1730537)
Supplement: Supplementary file 1 [file Data_Sheet_1.PDF]

Table S1: The level of anxiety, depression, breathlessness, overall health status, and quality of life among patients with PRISm (n =101)

**Anxiety**	**Frequency (%)**	**95% Conf. Interval**
**Abnormal or Borderline (defined as a HADS score of 8 to 21)**	**38 (37.6%)**	**28.6 to 47.5**
**Depression**	Frequency (%)	
**Abnormal or Borderline (defined as a HADS score of 8 to 21)**	**27 (26.7%)**	**18.9 to 36.3**
**Health status**	Frequency (%)	
**Medium to high impact of PRISm on health status (defined as an CAT score of 20 to 40)**	**45 (44.5%)**	**34.5 to 53.9**
**Breathlessness level**	Frequency (%)	
**Increased level of breathlessness (defined as an mMRC score of 2 to 4)**	**37 (36.6%)**	**27.1 to 45.9**
**Quality of life**	Frequency (%)	
**Impaired quality of life (defined as a SGRQ total score ≥ 25)**	**67 (66.3%)**	**56.4 to 74.9**

Abbreviations: HADS: hospital anxiety and depression scale, CAT: COPD assessment test, mMRC: modified medical research council, CAT: COPD assessment test, SGRQ: George's respiratory questionnaire.
